# Evaluation of Physicochemical and Antioxidant Properties of Peanut Protein Hydrolysate

**DOI:** 10.1371/journal.pone.0037863

**Published:** 2012-05-31

**Authors:** Lin Tang, Jie Sun, Hui Cui Zhang, Chu Shu Zhang, Li Na Yu, Jie Bi, Feng Zhu, Shao Fang Liu, Qing Li Yang

**Affiliations:** 1 Shandong Peanut Research Institute, Qingdao, People's Republic of China; 2 Shandong Normal University, Jinan, People's Republic of China; University of South Florida College of Medicine, United States of America

## Abstract

Peanut protein and its hydrolysate were compared with a view to their use as food additives. The effects of pH, temperature and protein concentration on some of their key physicochemical properties were investigated. Compared with peanut protein, peanut peptides exhibited a significantly higher solubility and significantly lower turbidity at pH values 2–12 and temperature between 30 and 80°C. Peanut peptide showed better emulsifying capacity, foam capacity and foam stability, but had lower water holding and fat adsorption capacities over a wide range of protein concentrations (2–5 g/100 ml) than peanut protein isolate. In addition, peanut peptide exhibited *in vitro* antioxidant properties measured in terms of reducing power, scavenging of hydroxyl radical, and scavenging of DPPH radical. These results suggest that peanut peptide appeared to have better functional and antioxidant properties and hence has a good potential as a food additive.

## Introduction

Peanut is the world's fourth most important source of edible vegetable oil and the third most important source of vegetable protein feed meal [1]. In China, peanuts have been grown as an oil seed crop for export and for production of the edible oil, while the protein residue in the form of oil cake is used as animal feed [2], [3]. Recently peanut protein has been receiving increasing attention from the food industry, as an additive in meat and dairy products, baked food, health food and other similar commercially important items. One of the notable features of peanut protein is its high nutritional value; but its functional properties, digestibility and bioactivity are relatively low [4]. However, its hydrolysate which is peanut peptide could have better physicochemical properties such as the solubility, emulsifying capacity, foam capacity, etc. Certain peptide sequences that are correlated with potent antioxidative and radical scavenging functions have been identified by sequence comparison of various proteins and are present in peanut protein [5].

The physiochemical properties of peanut peptide will play an important role when considering its application in food formulations and processing. To develop dietary quality peanut peptide for utilization as ingredients in the food industry, it is necessary to determine these properties of peanut peptide. Dong XH reported the possibility of improving the availability of Peanut protein isolate (PPI) by high-pressure homogenization (HPH) treatment via increasing extraction yield and enzymatic hydrolysis of the PPI, which can provide a better utilization of the peanut by-product [6]. As antioxidants are needed by our body to combat reactive oxygen species they are important as food additives. Hwang JY et al. reported for the first time that protein hydrolysates from defatted peanut kernels possess antioxidative activity [7]. It is hoped that the understanding of scavenging of radical capacity and other antioxidant properties of the peanut peptide may lead to use of peanut peptide as secondary foods especially for certain populations such as infants, the elderly and convalescing patients.

The present study was conducted to compare and contrast the biophysical properties of peanut protein isolate and its hydrolysed product, peanut peptide. Properties such as emulsifying capacity, foam capacity, turbidity, solubility, water holding and fat adsorption capacity were measured. The antioxidant properties of peanut peptide were also determined. To the best of our knowledge, these properties of peanut peptide have not been investigated so far.

## Materials and Methods

### Ethics statement

In this study, no specific permits were required for the described field studies. The study is not privately-owned or protected in any way. The field studies did not involve endangered or protected species.

### Materials

Defatted peanut protein powder was purchased from Shandong Tianshen Biological Protein Co., Ltd, China. Sodium dodecyl sulphate (SDS), and 1, 1-dipheny-2-picryhydrazyl (DPPH) were purchased from Sigma Chemical Co. (St. Louis, MO, USA). and All reagents were of analytical grade.

### Preparation of peanut peptides

Defatted peanut protein powder was added to alkali solution (pH9.0) to be alkali-soluble homogeneous solution, and was placed in the microwave extraction apparatus. The sample was extracted at constant ultrasonic power (300 W) and temperature (38°C) for a certain time (25 min) two times. After the reaction was complete, the reaction mixture was centrifuged. The supernatant was added to hydrochloric acid to adjust pH 4.5. The mixture was centrifuged and the precipitate was freeze-dried in order to obtain peanut protein isolated. The protein content is 95.1%. Peanut protein isolate (40 g) was mixed with distilled water (400 ml) in an Erlenmeyer flask (500 ml cap.) and held in a water bath at 90°C (Shanghai Yarong, China) for 20 min to inactivate the protein. The initial pH of the solution was adjusted to 8.0 using 0.1 M NaOH. The sample was subjected to enzymatic proteolysis by addition of papain (5000 u/g) in a reaction carried out at 50°C for 15 min in a microwave oven (XH-100A; Beijing Xianghu; China). The sample was held in a water bath at 100°C for 5 min to inactivate the enzyme. The reaction mix was centrifuged at 3500 *g* for10 min, the supernatant was recovered. The latter was concentrated in a rotary evaporator (RE52CS-1, Shanghai Yarong, China) at 50°C for 6 hours. The concentrate was freeze-dried (FD-1C-50, Beijing Boyikang, China) to obtain a powder of peanut peptides. The yield of peptide was 55% and the degree of hydrolysis was measured by OPA followed [8], and the degree of hydrolysis was 25.5%.

**Figure 1:**
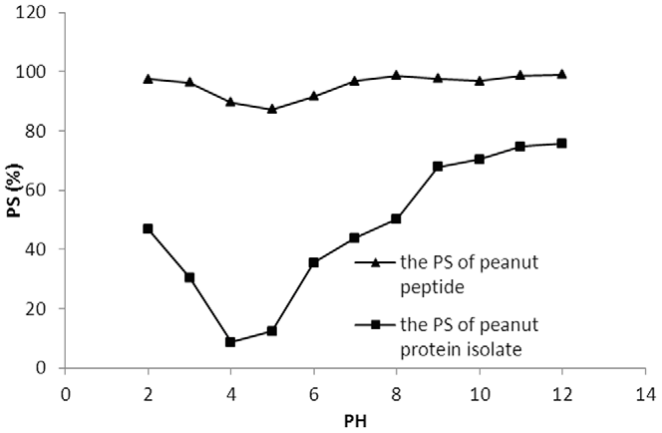
Protein solubility of peanut peptide (triangles) and peanut protein (squares) in the pH range of 2–12.

### Protein solubility (PS)

PS was determined according to the method of Tang et al. [9]. Protein samples were resuspended (0.1%w/v) in 0.01 M phosphate buffer adjusted to different pH values between pH 2 and 12. The protein solutions were stirred at room temperature for 30 min and then centrifuged (3500 *g*, 10 min). Then protein content in the supernatant was determined by the Kjeldahl method. The peanut solubility was calculated as follows:

PS (%)  =  (protein content of the supernatant/ initial total protein content of the sample) ×100

### Protein solution turbidity

Protein solution turbidity was determined according to the method of Benjakul [10]. Turbidity was measured as absorbance in a spectrophotometer at 500 nm using deionised water as the blank. To study the effect of pH, protein samples (200 mg) were resuspended in 100 ml phosphate buffer and the pH was adjusted between 2 and 12 as required. The solutions were stirred at room temperature for 30 min before measuring turbidity. To study the effect of temperature on protein solution turbidity, the same procedure was followed, except that the solutions were held at different temperatures between 30°C to 80°C for 20 min.

**Figure 2:**
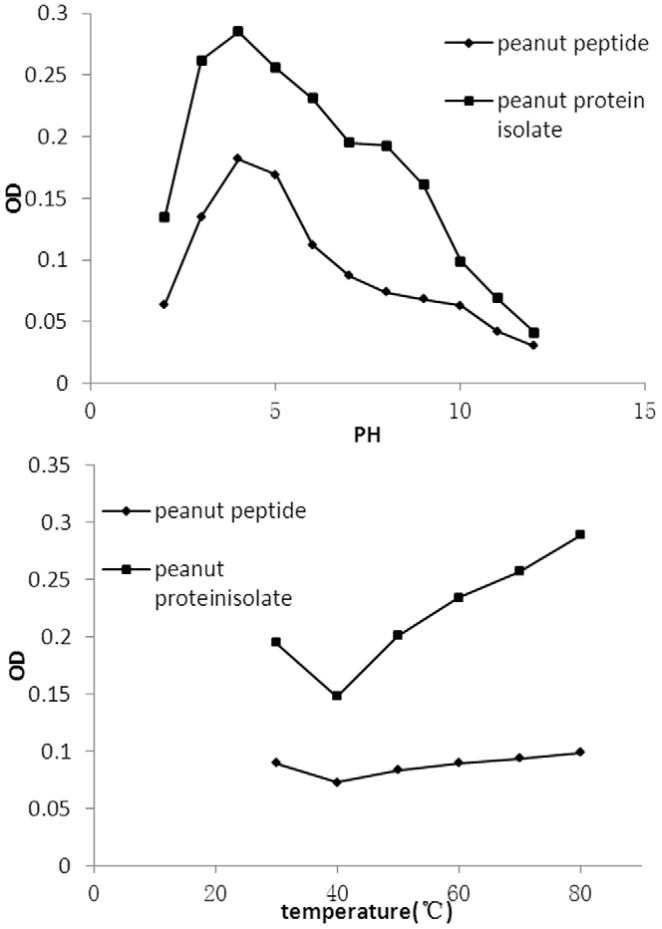
Effect of different pH (I) and heating temperatures (II) on turbidity of peanut peptide (diamonds) and protein (squares).

### Emulsifying Activity and Emulsion Stability (EA) and (ES)

EA and ES were determined by the turbidimetric method of Pearce and Kinsella [11]. Protein samples (20 ml) at 0.1–0.5% w/v in 0.1 M NaH_2_PO_4_- Na_2_HPO_4_ buffer, pH 8.0, were stirred at room temperature for 30 min and soybean oil (2 ml) was added to each. The mixtures were homogenized in HG-15D homogenizer (DAIHAN Scientific CO., Ltd. Korea) for 1 min at the maximum velocity. Immediately after homogenization and emulsion formation, 50-µL aliquots of the emulsion were taken at 0 and 10 min from the bottom of the tube and added to 5 mL of 0.1% sodium dodecyl sulphate (SDS) solution (1∶100 dilution). The diluted emulsion was shaken very briefly in a vortex mixer and the absorbance at 500 nm was read in the spectrophotometer (Ultra-spec2001, Biochrom, England). The EA and ES were calculated as follows:
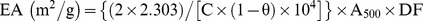
Where C is the initial concentration of protein; θ is the fraction of soybean oil used to form the emulsion; A is the absorbance and DF is the diluting factor (100).
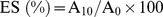



**Figure 3:**
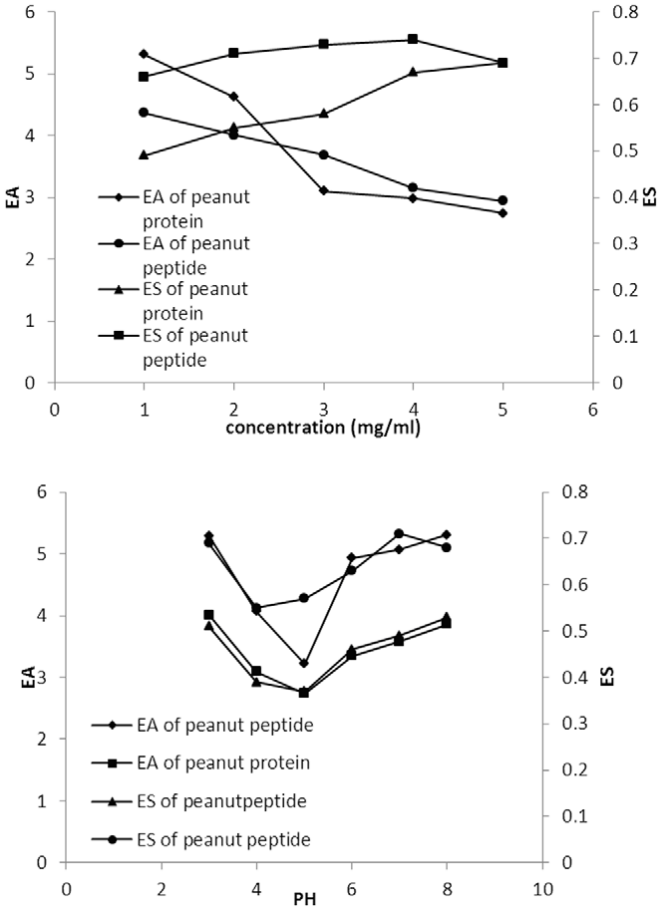
Effect of different protein concentrations (I) and pH (II) on EA and ES of peanut protein (diamonds and triangles) and peanut peptide (circles and squares).

### Foam capacity and foam stability

Foam capacity (FC) and foam stability (FS) were determined by the method of Agyare [12] and Arogundade [13]. Protein dispersions (10∼50 mg/ml, 40 ml) were stirred at room temperature for 30 min and then homogenized in HG-15D homogenizer (DAIHAN Scientific CO., Ltd. Korea) for 0.5 min at the maximum velocity. The solutions were transferred into 100 ml measuring cylinder and the volume of foam was recorded. The volume of foam was recorded again after 60 min. Foam capacity and foam stability were calculated by:
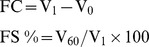
Where V_1_ is the total volume of the solution, V_0_ is the volume of the foam and V_60_ is the volume of the form measured after 60 min.

**Figure 4:**
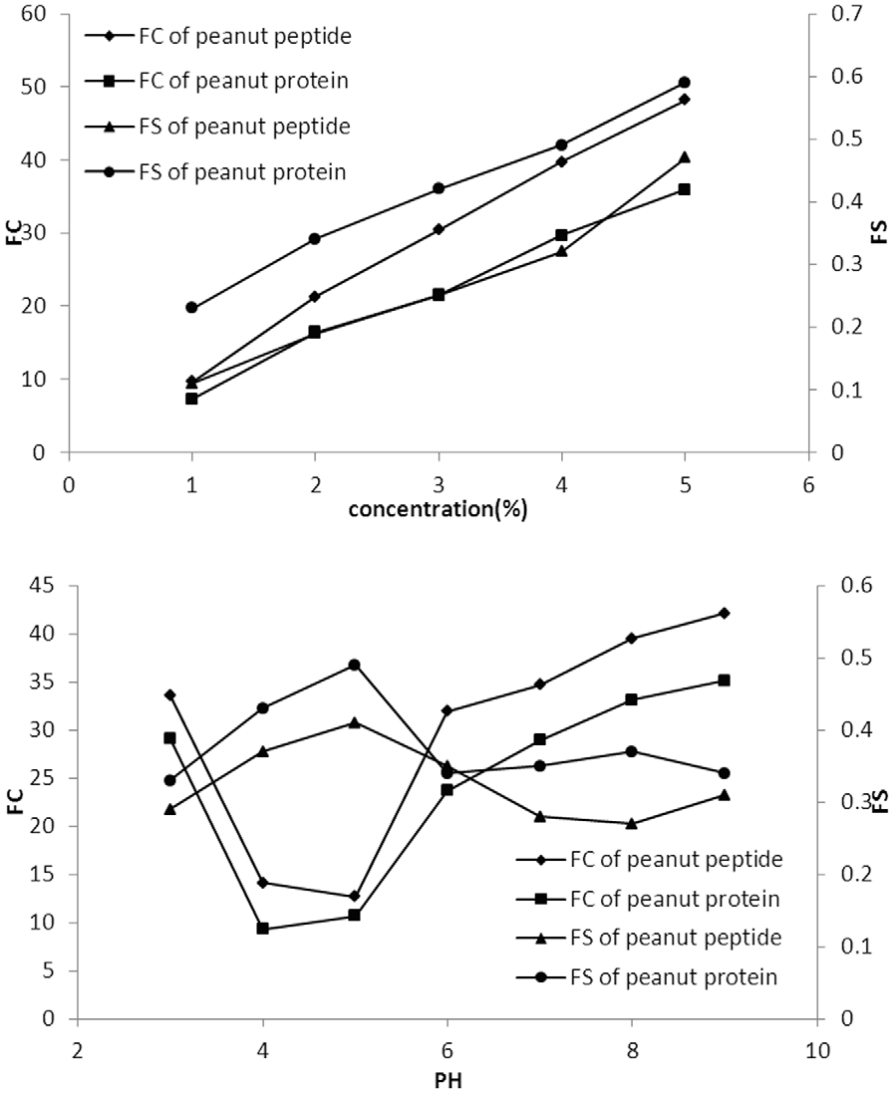
Effect of different protein concentrations (I) and pH (II) on FC and FS of peanut protein (squares and circles) and peptide (diamonds and triangles).

### Water holding capacities (WHC) and fat absorption capacities (FAC)

WHC and FAC were determined using the method of Ahmedna et al. [14] and Tomotake et al. [15], with slight modifications. Briefly, 5 g of protein sample was weighed into 50 ml pre-weighed centrifuge tubes. For each sample, distilled water was added to a series of tubes in small increments under continuous stirring with a glass rod, just enough to wet the samples. After the mixture was thoroughly wetted, samples were centrifuged (3500 g, 10 min). After the centrifugation, the amount of added distilled water resulting in the supernatant liquid in the test tube was recorded. WHC (grams of water per gram of sample) was calculated as follows:
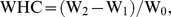
Where W_0_ is the weight of the dry sample (g), W_1_ is the weight of the tube plus the dry sample (g), and W_2_ is the weight of the tube plus the sediment (g). Triplicate samples were analyzed for each sample.

**Figure 5:**
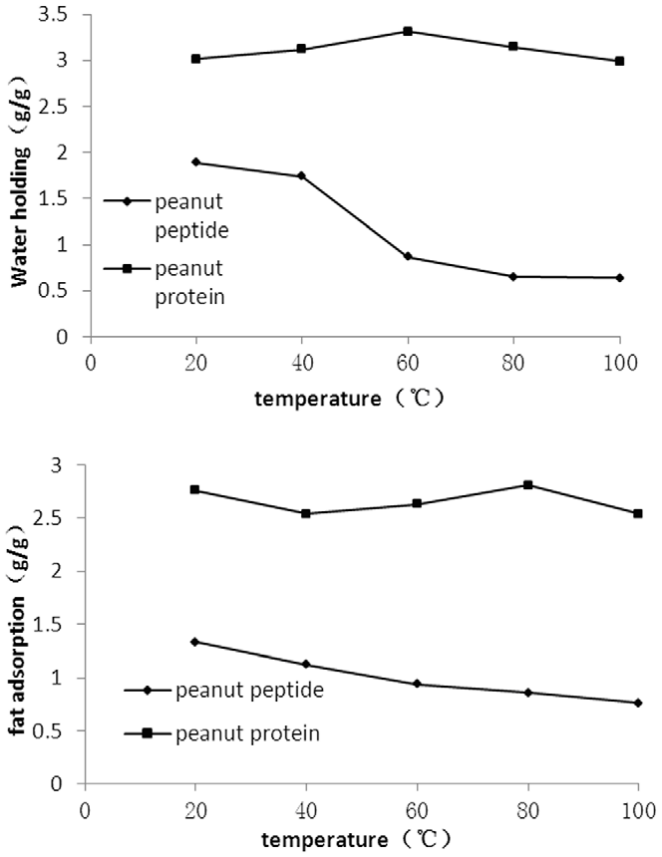
Effect of temperature on water holding capacity (I) and fat adsorption capacity (II) of peanut peptide (diamonds) and peanut protein (squares).

**Table 1:** Antioxidant properties of peanut peptide were measured by different biochemical tests as indicated.

concentration (mg/ml)	reducing power (OD)	hydroxyl radical scavenging (%)	DPPH radical scavenging (%)	antioxidant activities (%)
5	0.317±0.06	37.29±0.48	47.63±1.11	13.45±0.54
10	0.432±0.03	54.34±1.07	65.33±0.78	24.48±0.66
15	0.494±0.01	64.78±1.81	71.34±0.96	33.43±1.32
20	0.584±0.04	72.45±1.12	75.65±1.47	37.28±1.61
25	0.671±0.03	82.06±1.43	74.88±0.98	40.17±0.77

All samples were measured in triplicate and the values of mean± s.d. are presented.

For FAC, 1 g of sample was weighed into 50 ml pre-weighed centrifuge tubes and thoroughly mixed with 4 ml of corn oil. The protein–oil mixture was centrifuged (3500 g, 10 min). Immediately after centrifugation, the supernatant was carefully removed, and the tubes were weighed. FAC (grams of oil per gram of protein) was calculated as follows:
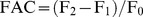
Where F_0_ is the weight of the dry sample (g), F_1_ is the weight of the tube plus the dry sample (g), and F_2_ is the weight of the tube plus the sediment (g). Triplicate samples were analyzed for each sample.

### Reducing power

Peptide solution, 0.5 ml was added to test tubes followed by 1.8 ml 0.1 M phosphate buffer solution (pH 6.6), 1.8 ml 1% potassium ferricyanide and the mixture was allowed to stand for 20 min at 50°C, and lastly 1.8 ml 10% trichloroacetic acid was added. The mixture was centrifuged (3000 g, 10 min). After centrifugation, 1.5 ml of the supernatant was transferred into another tube, followed by 1.5 ml distilled water, 0.5 ml 0.1% ferric trichloride. All spectrophotometer readings were performed at 700 nm using deionised water as the blank. The Fe^3+^ is reduced to Fe^2+^ in the presence of a reducing agent in the sample which causes a change in the colour of the solution, which can be detected spectrophotometrically at 700 nm.

### Measurement of DPPH scavenging activity

The DPPH scavenging activity of the extracts was measured by the method of Shimada et al. [16]. 1.5 ml of peptide solution was added to 1.5 ml of 0.1 mM of DPPH (in 95% ethanol). The mixture was allowed to stand for 30 min at room temperature before measuring the absorbance at 517 nm. The antioxidant activity was expressed as a percentage of scavenging activity on DPPH radical:
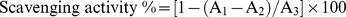
Where A_1_ is the absorbance of the DPPH and sample; A_2_ is the absorbance of the samples and 95% ethanol; A_3_ is the absorbance of the DPPH and distilled water.

### Hydroxyl radical scavenging

Each 0.5 ml peptide solution was added into test tubes followed by 0.5 ml of 9.1 mM salicylic acid-ethanol solution, 0.5 ml 9.1 mM FeSO_4_, 3.5 ml distilled water and 5 ml 8.8 mM H_2_O_2_. The absorbance was measured at 510 nm. Same mixture but without the protein sample was used as a blank.
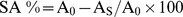
Where SA is the hydroxyl radical scavenging capacity; A_0_ is the absorbance of the blank; as is the absorbance of the sample.

### Antioxidative activity

Each 200 µl peptide solution was added into test tubes followed by 3 ml 0.1 M phosphate buffer solution (pH 7), 200 µl 50 mM linoleic acid-ethanol (95%); 50 µl 50 mmol/L FeCl_2_-EDTA and the mixture stand for two days at 50°C, and then 400 µl the mixture was transferred into another tube, followed by 200 µl 1 M FeCl_2_ and 1.0 M HCl; 200 µl 30% KSCN. All spectrophotometer readings were performed at 700 nm using same mixture but without the protein sample as the blank. The antioxidative activity was calculated as follows:
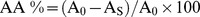
Where AA is the antioxidative activity; A_0_ is the absorbance of solution without peanut peptide; A_S_ is the absorbance of solution with peanut peptide

## Results and Discussion

### Peanut peptide and protein solubility

PS of a given protein is directly related to pH of the solution as it depends on the pI of the protein. Hence the PS of peanut peptide and peanut protein isolate were determined across a range of pH values in phosphate buffer (Fig. 1). The PS of peanut peptide was found to be comparatively stable across this wide pH range, with a minimum at pH 4–5 (Fig. 1). The peanut protein had an isoelectric point (IEP) at pH 4.5 as seen from its drastically reduced solubility at this pH; and its PS and increased gradually below pH 3 and above pH 6. While both peanut peptide and peanut protein isolate exhibited a similar trend in PS profile, the PS of the latter was comparatively much lower. Proteins are amphoteric molecules, so that in an acidic medium, at pH values lower than their pI, they behave as positive ions and repel each other, and thus have good dispersivity. As the pH value approaches pI, the solubility decreases. Similarly in alkaline solutions, at pH values is >pI, protein molecules change into negative ions, and the solubility increases [17]. These phenomena affect PS and the turbidity of the protein solution. In general, for most applications as food additives, good solubility across a wide pH range is a desirable attribute; and peanut peptide appears to be superior to peanut protein in this aspect.

### Protein solution turbidity

Turbidity of a protein solution is inversely related to its solubility, which in turn depends on the pH of the solution as discussed above. Accordingly, the turbidity of peanut peptide was the maximum at pH 4–5, where its PS was minimum, and the turbidity reduced gradually below pH 3 and above pH 6 (Fig. 2 (I)). Peanut protein isolate had a similar turbidity profile as peanut peptide (Fig. 2 (I)). The PS and therefore turbidity of a protein solution depend on the temperature also. PS of both peanut peptide and protein increased between temperatures 30–40°C as seen from reducing turbidity (Fig. 2 (II)). At higher temperatures both proteins began to aggregate and precipitate resulting in increased turbidity.

### Emulsifying activity and stability

EA and ES are important characteristics for a food additive and may limit its use. These properties were compared for peanut protein and peanut peptide over a range of protein concentrations and pH values (Fig. 3). For both, with the increasing protein concentration, the EA reduced, while the ES increased. The EA of peanut peptide is higher than that of protein, but the ES was lower (Fig. 3 (I)). In general, the PS of a protein has a significant effect on the EA and ES. Although the PS of peanut peptide was much greater than that of peanut protein isolate, the ES of peptide was lower than that of protein. These results were consistent with Guanli Zhao, and His experiments proved that the limited emzymatic hydrolysis improved the functional properties of peanut protein isolate, such as protein solubility and gel-forming ability, but impaired the emulsifying activity index [18]. A possible reason could be that the presence of insoluble protein may strengthen the stability of emulsion.

The EA and ES of peanut peptide were minimal near IEP or pH 4–5, and increased above pH 5 and below pH 4 (Fig. 3 (II)). It is suggested that the EA is related to the PS because the effects of pH on EA and ES are similar to those on PS. The EA values of peanut peptide were, by and large, significantly higher than those of peanut protein. The net charge on the protein molecule is zero at its IEP and the absorbed protein is rarely at the interfacial of hydrated layer and fat globules, so the EA and ES were minimal.

### Foaming Capacity (FC) and Foaming Stability (FS)

The FC and FS of both, peanut peptide and protein increased with the increasing protein concentration (Fig. 4 (I)). At all concentrations, the FC of peanut peptide was higher, and its FS was lower than that of peanut protein (Fig. 4 (I)). It has been reported that limited hydrolysis may improve foaming capacity but decrease foam stability [12]. The formation of protein-based foams involves the diffusion of soluble proteins towards the air-water interface and rapid conformational change and rearrangement at the interface. The FS requires formation of a thick, cohesive, and viscoelastic film around each gas bubble. The protein concentration affects the properties of the solution. The higher the protein concentration the thicker, more cohesive, and viscoelastic are the gas bubbles. When the concentration is high, protein molecules form thicker adsorbed films which is beneficial to FS [19]

The FC of both peanut peptide and peanut protein were minimum at pH 4–5, but the FS was maximum at this pH (Fig. 4 (II)). It is likely that a more compact structure of protein at pH values the IEP, results in low FC. On the other hand, because of the increasing of static charge near IEP, the surface hydrophobicity is weakened, causing an increased flexibility of the protein and thereby increased FS.

### Water holding and fat adsorption capacity

The WH and FAC of peanut peptide were lower than those of peanut protein isolate at all temperatures tested (Fig. 5), despite the fact that under these conditions the PS of peanut peptide was higher. Ahmedna et al. [14] suggested that high protein solubility was not necessarily related to high WH. The lower WH and FAC of peanut peptide may be related to its low molecular weight leading to a smaller interface surface area. These results agree with those reported by Cumby et al. [20]. The FAC of peanut peptide gradually reduced with increasing temperature. This is mainly due to the changes in viscosity of oil at different temperature as oil has high viscosity at low temperature.

### Antioxidant properties of peanut peptide

Reducing power, hydroxyl radical scavenging activity, DPPH radical scavenging activity and antioxidant were determined at different concentrations of peanut peptide in solutions and in general were found to increase with increasing protein concentration (Table 1). Reducing power is regarded as an important index in evaluation of an antioxidant. The absorbance values given in Table 1 reflect the reducing power of the peanut peptide solution: the higher the absorbance value the stronger is the reducing power [21]. Hydroxyl radicals are the most reactive among the reactive oxygen species as they react with almost all the cell components, which is deleterious [22]. The hydroxyl radicals (OH) scavenging capacity of peanut peptide was studied by the salicylic acid method. Lipid is the principal component of cell membranes. A cell is seriously damaged by the lipid peroxidation. Hence the inhibition of linoleic acid peroxidation is an important index of antioxidant properties. The model of scavenging stable DPPH radical is a widely used method to evaluate antioxidant activities in a relatively short time compared with other methods. The addition of the peanut peptide to the DPPH solution caused a rapid decrease in the optical density at 517 nm. The degree of discoloration indicates the scavenging capacity of the sample. The peanut peptide could effectively scavenge hydroxyl and DPPH radicals and was able to inhibit linoleic acid peroxidation. Results shown in Table 1 thus indicate that peanut peptide had overall good antioxidant properties. The 25 mg/ml peanut peptide showed higher reducing power, hydroxyl radical scavenging capacity, and DPPH radical scavenging capacity when compared to the other four concentrations. These results clearly show that high concentrations of peanut peptide had higher antioxidant power. Similar results have been found in many plants. Janet C C confirmed highest antioxidant activity was found in bean protein isolate hydrolysates [23]. Jean-Yu Hwang reported that protein hydrolysates obtained from defatted peanut kernels with esperase treatment for 2 h exhibited higher antioxidative activity toward linolenic acid peroxidation than peanut protein isolate [7]. A variety of beneficial effects for human health are present in the peanut peptides, and these peptides could be used in dietary supplement preparations, or as a food additive, to prevent oxidation in food products.

### Conclusion

Results presented here demonstrated that the solubility of the peanut peptide was greater than that of peanut protein over a wide range of pH values (2 to 12). Similarly, the turbidity of peanut peptide solution was significantly lower than that of protein isolate across a wide range of pH (2–12) and temperatures (30–80°C). Its water holding and fat adsorption capacities were lower than those of protein isolate. The peanut peptide showed better emulsifying capacity, foam capacity and foam stability over a large concentration range (2 g/100 ml to 5 g/100 ml). In addition, the peanut peptide exhibited in vitro antioxidant properties as determined in terms of reducing power, hydroxyl radical scavenging activity, antioxidant and DPPH radical scavenging activity. Taken together, these results suggest that peanut peptide has good prospects for application in the food industry.

## References

[1] Lusas EW (1979). Food uses of peanut protein.. Journal of the Asmerican Oil Chemist Society, 56,.

[2] Damame SV, Chavan JK, Kadam SS (1990). Effects of roasting and storage on proteins and oil in peanut kernels.. Plant Foods for Human Nutrition, 40,.

[3] Patil UG, Chavan JK, Kadam S, Salunkhe DK (1993). Effects of dry heat treatments to peanut kernels on the functional properties of the defatted meal.. Plant Foods for Human Nutrition, 43,.

[4] Zhang Y, Wang Q (2007). Peanut protein hydrolysis by Alcalase to prepare peanut oligopeptides.. Transaction of the CASE, 23,.

[5] Hettiarachchy NS, Griffin VK, Gnanasambandam R (1996). Prepartion and Functional Properties of a protein Isolated from Defatted Wheat Germ.. Cereal Chemistry, 73,.

[6] Dong XH, Zhao MM, Shi J, Yang B, Li J (2011). Effects of combined high-pressure homogenization and enzymatic treatment on extraction yield, hydrolysis and function properties of peanut proteins. Innovative Food Science and Emerging Technologies..

[7] Hwang JY, Shyu YS, Wang YT, Hsu CK (2010). Antioxidative properties of protein hydrolysate from defatted peanut kernels treated with esperase.. LWT - Food Science and Technology.

[8] Nielsen PM, Petersen D, Dambmann C (2001). Improved method for determining food protein degree of hydrolysis.. Journal of Food Science, 66,.

[9] Tang CH (2007). Functional properties and in vitro digestibility of buckwheat protein products: Influence of processing.. Journal of Food Engineering,.

[10] Benjakul S, Visessanguan W, Srivilai C (2001). Porcine plasma protein as proteinase inhibitor in big eye snapper (Priacanthus tayenus) muscle and surimi.. Journal of the Science of Food and Agriculture, 81,.

[11] Pearce KN, Kinsella JE (1978). Emulsifying properties of proteins: Evaluation of a turbidimetric technique.. Journal of Agricultural and Food Chemistry, 26,.

[12] Agyare KK, Addo K, Xiong YL (2009). Emulsifying and foaming properties of transglutaminase-treated wheat gluten hydrolysate as influenced by pH, temperature and salt.. Food Hydrocolloids, 23,.

[13] Arogundade LA (2006). Functional characterization of Tef (Eragostics tef) protein concentrate: Influence of altered chemical environment on its gelation foaming and water hydration properties.. Food Hydrocolloid, 20,.

[14] Ahmedna M, Prinyawiwatkul W, Rao RM (1999). Solubilized wheat protein isolate: Functional properties and potential food applications. Agric.. Food Chemistry, 47,.

[15] Tomotake H, Shimaoka I, Kayashita J, Nakajoh M, Kato N (2002). Physicochemical and functional properties of buckwheat protein product.. Journal of Agricultural and Food Chemistry, 50,.

[16] Shimada T, Fujikawa K, Yahara K, Nakamura T (1992). Antioxidant properties of xanthan on the autoxidation of soybean oil in cyclodextrin emulsion.. Journal of Agricultural and Food Chemistry, 40,.

[17] Yin YR, Liu WQ, Xiao KJ (1996). The study of Sesame protein solubility and emulsification.. Food Science, 17,.

[18] Zhao G, Liu Y, Zhao M, Ren J, Yang B (2011). Enzymatic hydrolysis and their effects on conformational and functional properties of peanut protein isolate.. Food Chemistry,.

[19] Akintayo E T, Oshodi A A, Esuoso KO (1999). Effects of NaCl ionic strength and pH on the foaming and gelation of pigeon pea (Cajanus cajan) protein concentrates.. Food Chemistry, 66,.

[20] Cumby N, Zhong Y, Naczk M (2008). Antioxidant activity and water-holding capacity of canola protein hydrolysates.. Food Chemistry, 109,.

[21] Pellegrini N, Proteggente A, Pannala A (1999). Antioxidant activity applying an improved ABTS^+^ radical cation decolorisation assay.Food Radical Biology and Medicine, 26,.

[22] Siddhuraju P, Becker K (2007). The antioxidant and free radical scavenging activities of processed cowpea (Vigna unguiculata (L.) Walp) seed extracts.. Food Chemistry, 101,.

[23] Janet CC, Alan Javier HA, Cristian JM, Carmen JH, Manuel A, Julio G C, Javier V, Gloria D O (2012). Antioxidant and metal chelating activities of *Phaseolus vulgaris* L. var. Jamapa protein isolates, phaseolin and lectin hydrolysates.. Food Chemistry,.

